# Colorimetric Biosensors: Advancements in Nanomaterials and Cutting-Edge Detection Strategies

**DOI:** 10.3390/bios15060362

**Published:** 2025-06-05

**Authors:** Yubeen Lee, Izzati Haizan, Sang Baek Sim, Jin-Ha Choi

**Affiliations:** School of Chemical Engineering, Clean Energy Research Center, Jeonbuk National University, 567 Baekje-daero, Deokjin-gu, Jeonju-si 54896, Jeonbuk State, Republic of Korea; ybdona@jbnu.ac.kr (Y.L.); izzatihaizan22@jbnu.ac.kr (I.H.); harry1313@jbnu.ac.kr (S.B.S.)

**Keywords:** colorimetric biosensors, nanomaterials, nanozymes, visual detection, point-of-care testing

## Abstract

Colorimetric-based biosensors are practical detection devices that can detect the presence and concentration of biomarkers through simple color changes. Conventional laboratory-based tests are highly sensitive but require long processing times and expensive equipment, which makes them difficult to apply for on-site diagnostics. In contrast, the colorimetric method offers advantages for point-of-care testing and real-time monitoring due to its flexibility, simple operation, rapid results, and versatility across many applications. In order to enhance the color change reactions in colorimetric techniques, functional nanomaterials are often integrated due to their desirable intrinsic properties. In this review, the working principles of nanomaterial-based detection strategies in colorimetric systems are introduced. In addition, current signal amplification methods for colorimetric biosensors are comprehensively outlined and evaluated. Finally, the latest trends in artificial intelligence (AI) and machine learning integration into colorimetric-based biosensors, including their potential for technological advancements in the near future, are discussed. Future research is expected to develop highly sensitive and multifunctional colorimetric methods, which will serve as powerful alternatives for point-of-care testing and self-testing.

## 1. Introduction

Biosensors have made significant progress with the development of nanomaterials and the innovation of application technology [1,2,3]. The early generation of biosensors relied on simple enzyme-based or antibody-based systems, but they are now developing into ultrasensitive biosensors that combine various high-quality nanomaterials, such as metal nanoparticles, graphene, and nanoplasmonic structures [4,5]. Many different nanomaterials are used in the study of biosensors, specifically for colorimetric biosensors [6]. Colorimetric biosensors can detect reactions with specific analytes through color changes and make simple as well as instantaneous diagnostic methods possible [7,8,9].

With the increasing demand for fast and cost-effective detection methods, colorimetric biosensors are receiving much attention due to their unique properties, such as simple visual detection, high sensitivity, and minimization of device dependence. Specifically, in clinical and on-site fields, an immediate, highly sensitive, and affordable sensing system is imperative for diagnosis. However, until now, achieving the stated criteria has remained a key challenge [10,11]. As an alternative, point-of-care testing (POCT) allows for rapid diagnosis and treatment decisions in areas that lack infrastructure; thus, they can facilitate a rapid diagnosis for the early detection and isolation of infectious diseases [12,13]. Moreover, colorimetric biosensors can streamline chronic disease management by reducing time, manpower, and medical costs compared to laboratory testing. Traditional analysis methods provide high precision but require complex equipment with high costs and long analysis times, which limit their use in POCT [6,14]. Colorimetric biosensors, on the other hand, can rapidly and easily detect target analytes using an intuitive signaling mechanism called color change.

Recently, nanoplasmonic colorimetric biosensors have dramatically improved their sensitivity for the detection of trace amounts of analytes by utilizing optical properties such as local surface plasmon resonance (LSPR) [15]. LSPR-based colorimetric research primarily involves noble metal nanoparticles, such as gold (Au) and silver (Ag). In particular, the sensitive LSPR effect is being studied by altering the size, shape, and surrounding environment of these nanoparticles [16]. In addition, researchers are focusing on the use of nanomagnets in colorimetric biosensors, which are reported to be more stable and reproducible than conventional protein enzymes. Nanomagnets are actively researched due to their advantages, which include higher stability, lower production costs, various catalytic functions, and longer shelf life compared to conventional enzymes [17]. Ultimately, proving a nanotechnology-combined colorimetric biosensor is expected to enhance sensitivity and selectivity, which enables the detection of trace amounts of analytes. This could further improve POCT and increase the access to medical care in underdeveloped areas by providing quick results with simple equipment [18,19].

Although various sensitivity enhancement techniques have been developed, the colorimetric method still has limitations of being less sensitive and having interference in quantitative analyses compared to other sensing techniques [20]. In order to overcome this limitation, research is actively conducted to integrate and utilize the colorimetric method and other signal conversion methods, such as fluorescence, electrochemical, and surface-enhanced Raman spectroscopy (SERS) signal enhancement methods [21,22,23]. These complex sensing techniques not only improve the sensitivity of the colorimetric method but also enable real-time monitoring and more precise quantitative analyses [24]. In particular, a dual sensing system improves accuracy by combining color change in a colorimetric analysis with other enhancement signals, such as fluorescence and electrochemical signals, for a refined quantitative analysis [25,26].

Moreover, a colorimetric analysis using smartphone cameras provides more accurate results by quantitatively analyzing red, green, and blue (RGB) values [27,28,29]. In recent years, the integration of machine learning and artificial intelligence (AI) platforms into colorimetric methods has further improved analytical precision and automated data interpretation. AI is addressing the limitations of traditional colorimetric methods by learning RGB values and color change patterns [30]. These smart devices and AI-based analysis technologies are accelerating the development of next-generation biosensors, such as wearable healthcare, rapid diagnosis, and smartphone-linked biosensors [31,32]. In the future, biosensors with higher sensitivity, reliability, and portability will be developed through convergence with AI. It is expected that personalized medical care and real-time monitoring will then be implemented more precisely.

Despite the advantages of nanomaterial-based colorimetric biosensors for their high sensitivity and easy visual reading, there are still several limitations in clinical applications. Most studies remain at the laboratory level and face various limitations, such as complexity in real biological environments, reducing reproducibility of clinical samples, and the possibility of biological interference in nanomaterials [33,34,35]. In addition, the absence of standardized clinical verification protocols, the instability of material synthesis, and the lack of proactive engagement between material scientists and clinicians are also major factors that hinder clinical translation [36,37,38,39,40]. To solve these problems, an integrated collaboration system from basic research to clinical verification and participation in the initial design stage of clinical trials is essential.

This review provides an overview of the working principles of colorimetric biosensors for analytical and detection applications, nanoparticle-based methods, and recent developments. It will specifically outline interaction principles such as aggregation, redox reactions, and shifts in plasmonic resonances. In addition, studies highlighting the roles of nanoparticles, including Au, Ag, and quantum dots (QDs), in colorimetric methods will be discussed. Finally, this review will summarize future prospects for colorimetric methods, with an emphasis on sensitivity improvement strategies through the incorporation of digital devices and AI.

## 2. Principles of Colorimetric Sensing

Fluctuations in the chemical composition or surrounding environment of nanoparticles can lead to changes in their optical properties and color [41]. In some colorimetric sensors, the color change is induced by the LSPR phenomenon, where free electrons around specific metal nanoparticles, such as Au or Ag, oscillate collectively in response to incident light [42]. The resonance conditions of LSPR can be adjusted based on factors such as size, shape, composition, refractive index change in the surrounding medium, and interactions between nanoparticles. These factors lead to color changes, which are characterized by alterations in the absorption and scattering spectra [43,44,45]. Nevertheless, apart from LSPR, colorimetric reactions can also be induced by other mechanisms, depending on the sensor design and the nanomaterials used [46]. For example, strategies such as color change via oxidation and reduction reactions of peroxidase, pH variation, and interactions with ions or biomolecules are commonly employed in colorimetric biosensors [47,48,49]. This section of the review will further clarify the optical and chemical principles underlying colorimetry. A detailed list of the principles of colorimetric biosensors is presented (Table 1).

### 2.1. LSPR-Based Colorimetric Biosensors

When specific metal nanoparticles are exposed to light, their free electrons resonate with the external electric field and oscillate. During this process, the collective oscillation of electrons inside the nanoparticle at a particular frequency causes strong absorption and scattering at a specific wavelength. This resonance phenomenon represents LSPR, which depends on the nanoparticle’s size, shape, and the refractive index of the surrounding medium [50,51]. Due to its sensitivity to the surrounding environment, LSPR is utilized in various biosensing platforms for signal amplification and colorimetric detection [52]. LSPR-based sensors have attracted attention due to their high sensitivity and simple analytical procedures, wherein, depending on how the LSPR changes, they can be classified into aggregation-based sensors and refractive index-based sensors [46].

**Figure 1:**
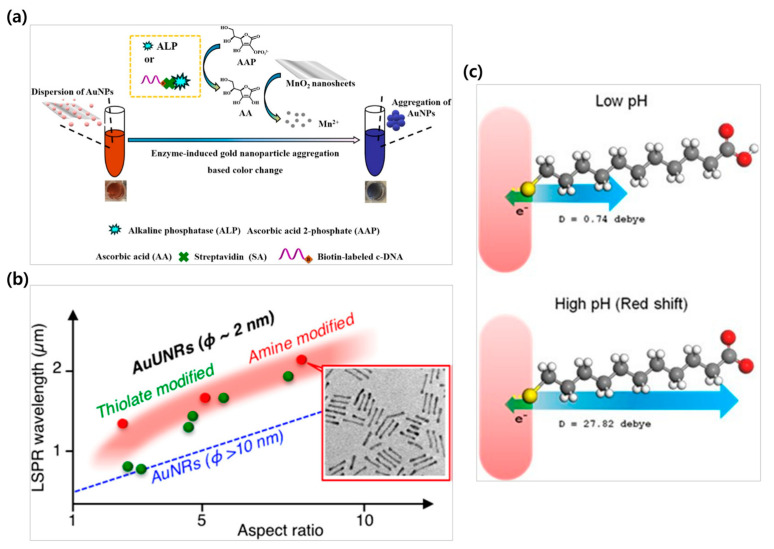
LSPR-based colorimetric biosensors. (**a**) A colorimetric sensing system based on enzyme-induced gold nanoparticle aggregation to detect the activity of alkaline phosphatase (ALP). Reproduced with permission from [56]. (**b**) Schematic illustrating the LSPR wavelength as a function of the aspect ratio for ultrathin gold nanorods. Reproduced with permission from [79]. (**c**) Representation of the electron-pulling force on GNR-MUA, inducing a blue or red wavelength shift of LSPR at low and high pH, respectively. Reproduced with permission from [80].

In the field of colorimetric biosensors, color changes due to the aggregation of noble metal nanoparticles are commonly utilized [51,52,53]. It mainly comprises colloidal noble metal nanoparticles, and aggregation between the nanoparticles is induced through specific interactions with target molecules [46]. Moreover, as the interparticle distance decreases, near-field electromagnetic coupling causes the LSPR peak to shift toward longer wavelengths, which results in a visible color change of the solution [54,55]. For example, colorimetric biosensors that utilize the color change from red to purple due to Au nanoparticle aggregation have been studied (Figure 1a) [52,56]. The visible color change not only enhances the sensor’s sensitivity but also enables naked-eye detection, which makes it suitable for POCT without the need for sophisticated instruments [57].

On the other hand, refractive index-based sensors involve the arrangement of metal nanoparticles on a fixed substrate, followed by the immobilization of biomolecules on their surfaces. In short, the binding with target molecules causes a change in the local refractive index around the nanoparticles [46,58]. Upon binding, the LSPR conditions change, and the signal is quantitatively measured by the movement of the absorption spectrum or the change in intensity [59,60,61]. In other words, LSPR-based sensors are not necessarily adapted for their ability to produce a color change [62]. Overall, LSPR is an optical phenomenon that is highly sensitive to changes in electron density distribution and the local refractive index on the surface of metal nanoparticles. These signals can be detected in various ways, including measurements of absorbance intensity, resonance wavelength shift, scattering intensity, and color changes [60,63]. As a result, LSPR-based sensors are widely used in both colorimetric and spectroscopy-based quantitative analyses [64].

In colorimetric methods, plasmonic nanoparticles, including Au, Ag, and alloys, are integrated based on the experimental design, as each metal has unique plasmonic properties with distinct advantages and disadvantages [65,66,67]. Advances in technology have enabled the fabrication of plasmonic metal nanostructures in nanometer sizes with specific shapes. [68,69]. The fabrication of metal nanoparticles is crucial, as the shape directly affects the resonance conditions and optical response of the nanoparticles [70]. For example, an increase in highly curved regions, such as sharp tips, leads to strong local electric field enhancement in response to external electromagnetic fields, which promotes the charge separation of free electrons [71]. Several forms of Au nanoparticles have been reported, including gold nanospheres, gold nanorods (AuNRs), and gold nanobipyramids (AuNBPs). Typically, gold nanostars (AuNSs) exhibit the lowest LSPR sensitivity—mainly due to the lack of sharp edges or tips [72]. In a study, Liebtrau et al. analyzed LSPR and electron–photon interactions with AuNSs with 50 nm cores and 3 nm tips. The contrasting roles of the core and the tip in the plasmonic nanostructures confirm the formation of the strongest electric field at the tip experimentally [73]. While the tips of AuNSs can positively affect the plasmonic properties, in general, caution should be taken when synthesizing the nanoparticles because AuNSs are very unstable [74].

Moreover, the symmetry of the nanoparticles affects the consistency and resonance efficiency of collective electron oscillations, which directly relates to LSPR signal strength [75]. In short, nanoparticle structures with a high symmetry allow for a strong electric field concentration. Depending on the polarization of incident light in a specific direction, the collective oscillations of electrons under resonance conditions are more aligned and remain stable. This, in turn, increases scattering and absorption signals [76]. Thus, a structure with high symmetry exhibits a stronger LSPR response and can contribute to the improvement of sensor sensitivity [77].

Finally, changes in the surrounding environment of the nanoparticles also affect the LSPR phenomenon [46,78]. Takahata et al. synthesized ultrathin gold nanorods (AuUNRs) with diameters below 2 nm by slowly reducing gold using the surfactant oleylamine (OA). As the aspect ratio (AR) of the nanorods increased, the LSPR exhibited a red shift. Moreover, when the surface ligand was changed from OA to glutathione (GSH) or dodecanethiol (C_12_SH), a blue shift in the LSPR was observed (Figure 1b) [79].

Toh et al. studied the effect of pH changes on LSPR, wherein after functionalizing the surface of AuNRs with 11-mercaptoundecanoic acid (11-MUA), a redshift of approximately 10.5 nm occurred at a high pH due to deprotonation of the carboxyl group (-COOH). In contrast, the neutral control group did not respond to pH changes, which confirms the suitability of this system for pH detection (Figure 1c) [80]. In conclusion, this section deals with the principles of LSPR phenomena and the properties of nanoparticles influencing them as a basis for understanding the operating principles of colorimetric biosensors.

### 2.2. Redox Reaction-Based Colorimetric Biosensors

Colorimetry is an analytical technique that quantitatively determines the presence or concentration of specific substances by observing color changes in a sample. The method uses not only the principles of LSPR but also various other colorimetric mechanisms [81,82,83]. The commonly used mechanism is based on redox reactions, where a substrate undergoes a color change as it is either oxidized or reduced [84]. A redox reaction is an electron-transfer process where one molecule is oxidized by losing electrons and another is reduced by gaining electrons [85].

HRP enzymes are the most widely used enzymes that induce the oxidation of substrates [86,87]. HRP utilizes H_2_O_2_ as an electron acceptor to receive and oxidize electrons from substrates. The results of this reaction can be visually confirmed, as the oxidized substrates cause a color change [88]. Similarly, there are many studies on glucose detection based on colorimetric methods, where glucose is oxidized by glucose oxidase to generate H_2_O_2_, which is then used by peroxidase (or peroxidase-mimicking nanozymes) to catalyze a color-producing reaction [89,90]. Enzyme-based colorimetric reactions are highly sensitive to experimental conditions, such as pH, temperature, and catalyst concentration, all of which significantly alter the efficiency and accuracy of the reaction [91,92].

**Figure 2:**
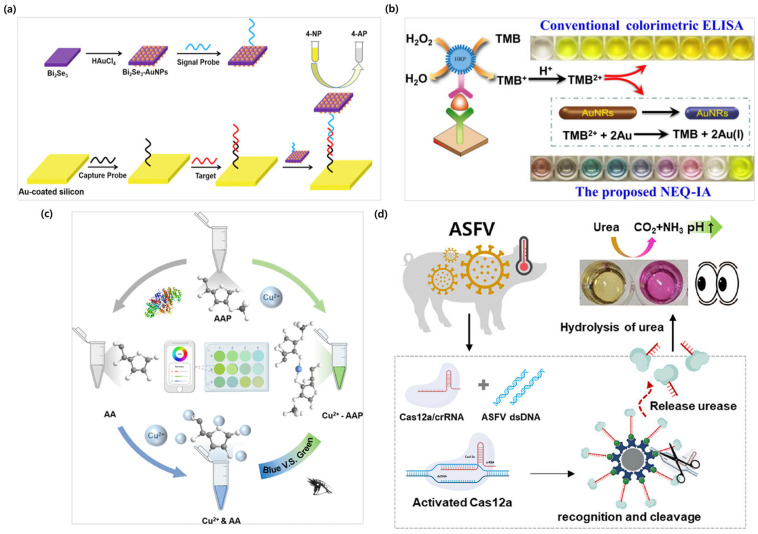
Redox reaction-based colorimetric biosensors. (**a**) Colorimetric detection of BRCA1 mutation using Bi_2_Se_3_-AuNPs, which catalyze the reduction of 4-nitrophenol (4-NP) to 4-aminophenol (4-AP). Reproduced with permission from [100]. (**b**) Colorimetric mechanism of the proposed NEQ-IA based on AuNR oxidation by TMB^2+^, which enables multicolor display. Reproduced with permission from [96]. (**c**) Colorimetric sensing system utilizing the coordination chemistry between ascorbic acid 2-phosphate (AAP) and copper ions. Reproduced with permission from [108]. (**d**) CRISPR/Cas12a-based system that uses the enzyme urease for the accurate and sensitive detection of ASFV. Reproduced with permission from [110].

The most widely used substrates in redox-based colorimetric analyses include TMB and ABTS (2,2′-azino-bis (3-ethylbenzenine-6-sulfonic acid) [93,94]. These compounds act as electron donors in redox reactions and are converted into colored products with specific absorbance peaks upon oxidation. TMB, for instance, is a colorless reduced form chemical that, in the presence of HRP and H_2_O_2_, is oxidized to a blue-colored intermediate, which can further be converted into a yellow oxidized form using excess oxidants. The absorbance is typically measured at 652 nm or 450 nm, and the intensity correlates with the degree of substrate oxidation (Figure 2b) [95,96]. ABTS also shows a blue-green color upon oxidation and is known for providing faster and more stable colorimetric reactions [97,98].

In the study of Chen et al., AuNPs, by removing electrons and protons from glucose, convert glucose into gluconic acid, and the removed electrons follow the dehydrogenation reaction pathway delivered to electron acceptors, such as oxygen or ABTS^+^• [89]. As a practical application of redox-based colorimetric reactions, Deshmukh et al. demonstrated that eco-friendly synthesized gold nanoparticles with gallnut extract (GNE-based AuNPs) have the multienzyme mimetic activity of glucose oxidase and peroxidase. These AuNPs first perform oxidase-mimicking reactions, which oxidize glucose into gluconic acid and H_2_O_2_ using oxygen as an electron acceptor. Then, the end product of glucose oxidation, H_2_O_2_, oxidizes TMB by peroxidase-mimicking reactions to produce blue-colored oxidized TMB (oxTMB) [99].

As another example, Bai et al. conducted a BRCA1 gene mutation detection study by focusing on the catalytic activity of bismuth selenide–gold nanoparticle (Bi_2_Se_3_-AuNP) nanocomposites that combine AuNPs and Bi_2_Se_3_ nanomaterials. In their study, Bi_2_Se_3_-AuNPs catalyze the reduction of 4-nitrophenol(4-NP) to 4-aminophenol(4-AP), wherein the Bi_2_Se_3_ nanosheets are easily oxidized to topological insulators, while the AuNPs supported on their surface can effectively transfer electrons due to AuNPs being excellent electron transfer agents, further promoting the reduction reaction of 4-NP and amplifying the colorimetric signal significantly. It can be seen that the catalytic activity of Bi_2_Se_3_-AuNP changes the color of 4-NP from bright yellow to colorless with a UV–vis spectrum change [100].

The redox principle described above also explains the colorimetric mechanism of nanozymes, which mimic natural enzyme activities [101,102]. Natural enzymes have undesired properties such as low stability, easy denaturation and deactivation, high production cost, and difficulty to recycle. In contrast, nanozymes have gained attention in biosensors and diagnostic applications due to their structural stability, low cost, tunable catalytic activity, and ease of surface modification [103,104]. Nanozymes can be categorized into various types, such as carbon-based, metal-based, metal oxide- or sulfide-based, metal–organic framework (MOF)-based, covalent–organic framework (COF)-based, and single-atom nanozymes [105]. For example, Liu et al. developed a pesticide detection system that employs the oxidase-mimetic catalytic activity of cerium-based coordination polymer nanoparticles (CPNs(IV)). These CPNs(IV) oxidized TMB, which produced a blue-colored signal [21].

In addition to redox-based mechanisms, hydrolysis, pH changes, and coordination complex formation are also employed in colorimetric biosensing [83,106,107]. For instance, ALP induces color changes by hydrolyzing phosphorylated substrates rather than undergoing direct redox reactions (Figure 2c) [106,108]. The enzyme binds to the substrate and forms colored products that enable colorimetric detection [108]. pH-based colorimetric detection uses pH indicators that change color in response to acidity or alkalinity, which provides a simple visual readout [109]. For example, phenol red appears yellow in low pH and red in high pH conditions, which makes it ideal for detecting biochemical reactions that involve pH changes (Figure 2d) [110]. Another example involves the use of Zn^2+^ ions and the ligand 5-Br-PAPS (5-bromo-2-pyridylazo-5-diethylaminophenol), which form a magenta-colored coordination complex, enabling the visual detection of zinc ion concentrations [111]. This section discusses the basic principles of oxidation–reduction reactions that act as signal generation mechanisms for colorimetric biosensors and how they work. In subsequent sections, we will further explore examples of colorimetric principles based on LSPR and redox reactions using various nanoparticles.

## 3. Nanomaterials-Based Colorimetric Biosensors

### 3.1. LSPR-Based Colorimetric Sensing

In recent research on colorimetric biosensors, various nanoparticles have been used as signal probes [81,112,113]. Because LSPR is sensitive to small environmental changes, the use of noble metal nanoparticles, such as Au and Ag, allows for fast, simple, and highly sensitive sensing [114]. Apart from noble metal nanoparticles, 2D nanomaterials and LSPR can be combined and used as optical probes for biosensors. The 2D nanomaterials feature atomic-scale thickness with unique electronic, optical, and mechanical properties [115]. The synergy between 2D nanomaterials and LSPR was shown to improve optical sensing [116]. In this section, we will discuss recent studies on colorimetric biosensors utilizing various noble metal nanoparticles and 2D nanomaterials based on LSPR principles.

#### 3.1.1. Noble Metal-Based Colorimetric Biosensors

In recent studies on colorimetric biosensors, various types of nanoparticles were actively utilized as probes for signal amplification and detection [117]. Li et al. developed a simple and portable Au-on-Au tip sensor for the specific, sensitive, and colorimetric detection of Salmonella Typhimurium. This sensor utilized the enzymatic cleavage reaction of Salmonella Typhimurium RNase H2 (STH2), which cleaves explicitly a nucleotide sequence. More specifically, the researchers linked DNA1 to an Au-coated layer inside a pipette tip and connected AuNP-DNA2 via a newly synthesized nucleic acid probe (NAP). In the presence of Salmonella, STH2 cleaved the NAP, which released AuNPs from the pipette tip and generated a visible colorimetric signal. This sensor is reusable and demonstrated a detection sensitivity of 3.2 × 10^3^ CFU/mL for Salmonella [118].

The size and shape of the nanoparticles used for the LSPR-based detection influence the sensor’s sensitivity, so these factors must be carefully considered [119]. For example, silver nanoprisms (AgNPRs) are highly attractive due to their unique plasmonic and optical properties, which differ from those of conventional Au and Ag nanoparticles commonly used in colorimetric biosensing applications. Hence, Li et al. developed a rapid, sensitive, and selective colorimetric detection platform for biothiols in human serum by using AgNPRs. Target biothiols form Ag-S covalent bonds on the AgNPR surface, preventing the etching by chloride ions (Cl^−^) present in human serum, which results in a blue-violet color.

**Figure 3:**
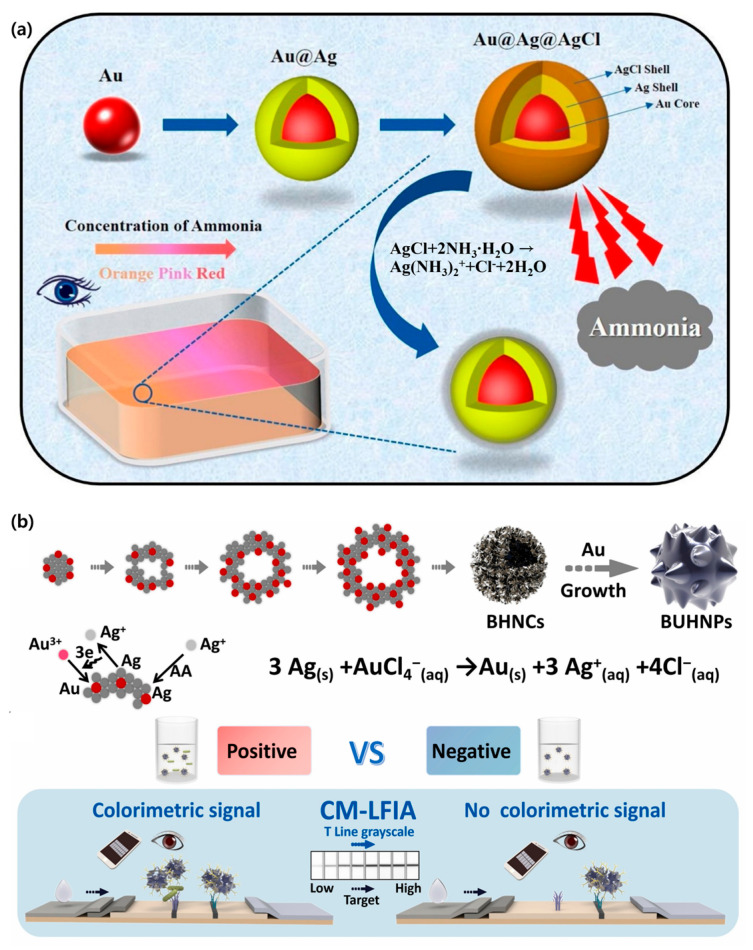
Noble metal-based colorimetric biosensors. (**a**) Schematic illustrating the Au@Ag@AgCl NP synthesis used for the colorimetric detection of NH_3_, wherein the Au core regulates the colorimetric sensor’s performance, while the Ag shell improves the stability of the nanoparticles entirely. Reproduced with permission from [125]. (**b**) Synthesis and characterization of bimetallic Ag–Au urchin-like hollow nanospheres (BUHNPs) by co-reduction and galvanic replacement for the detection of pathogenic bacteria via colorimetric LFIA. The BUHNP-CM-LFIA exhibits excellent colorimetric performance due to the distinctive hollow structure of the bimetallic nanocores and the heterogenous branched structure of BUHNP. Reproduced with permission from [126].

In contrast, in the absence of biothiols, the AgNPRs underwent an etching process that led to a color change from blue-violet to yellow. The results indicate the presence or absence of biothiols. The LOD of biothiols, achieved through this platform, was 0.041 µM [120]. Au and Ag nanoparticles are predominantly used in optical colorimetric methods, and their plasmonic resonance properties vary depending on their shape and size, which enables diverse sensing applications [121,122,123]. Sensitive colorimetric sensors can be developed using LSPR in AgNPs. However, AgNPs suffer from instability due to surface oxidation and Ag^+^ ion release, which decreases their effectiveness as sensing materials [124]. To overcome this limitation, Qiu et al. designed and utilized Au@Ag@AgCl core–shell nanoparticles. By decorating Ag into AgCl, the group improved the stability, while positioning Au at the core helped maintain the sensor performance (Figure 3a). The selective etching of AgCl and the Ag shell by NH_3_ exposed the underlying Au and Ag, which induces LSPR signal changes and allows colorimetric detection through the naked eye. The LOD of NH_3_ obtained in this study was 6.4 µM (approximately 14.3 ppm), which is significantly lower than in other studies [125].

Based on published research, colorimetric lateral flow immunoassays (CM-LFIAs) have desirable properties, such as simplicity, portability, rapid results, and cost-effectiveness, that make them suitable for early disease diagnosis, environmental monitoring, and food-safety testing. However, AuNPs, which are commonly used in CM-LFIA, exhibit insufficient colorimetric signal brightness [126]. Alloyed nanoparticles have gained significant attention due to their unique optical and photothermal properties [127]. To address this problem, Zhang et al. developed alloyed bimetallic Ag–Au urchin-like hollow nanospheres (BUHNPs). BUHNPs exhibit superior colorimetric signal brightness, strong photothermal signals, and high antibody binding efficiency (Figure 3b). Based on BUHNPs, a colorimetric LFIA (BUHNP-CM) was developed for the sensitive detection of pathogenic bacteria. The quantified LOD of this colorimetric system was 2.48 × 10^3^ CFU/mL—approximately four times lower than that of conventional AuNPs-LFIA (9.92 × 10^3^ CFU/mL) [126]. As demonstrated, optical probes for colorimetric signals can indeed induce spectral and color changes depending on the composition and ratio of the nanoparticles. In this section, the operation principle and performance improvement strategy were examined, focusing on various colorimetric biosensor research cases using LSPR nanoparticles, and these cases suggest the practical applicability and advancement direction of the technology. A detailed list of colorimetric biosensors with noble metal-based nanoparticles is presented (Table 2).

#### 3.1.2. Noble Metal-Decorated 2D Nanomaterial-Based Colorimetric Biosensors

Since 2D materials have desirable properties, such as a unique electronic structure, strong light absorption, and high mobility, their combination with metal nanoparticles can further enhance the sensitivity of LSPR [128]. Specifically, changing the structure of spherical silver nanoparticles (AgNPs) to 2D-like silver nanoparticles alters the LSPR response. The spherical AgNPs show a plasmon peak near 400 nm, which means that the LSPR peak is red-shifted via shape transformation to the nanoplates, underlining the importance of the 2D structure [129].

**Figure 4:**
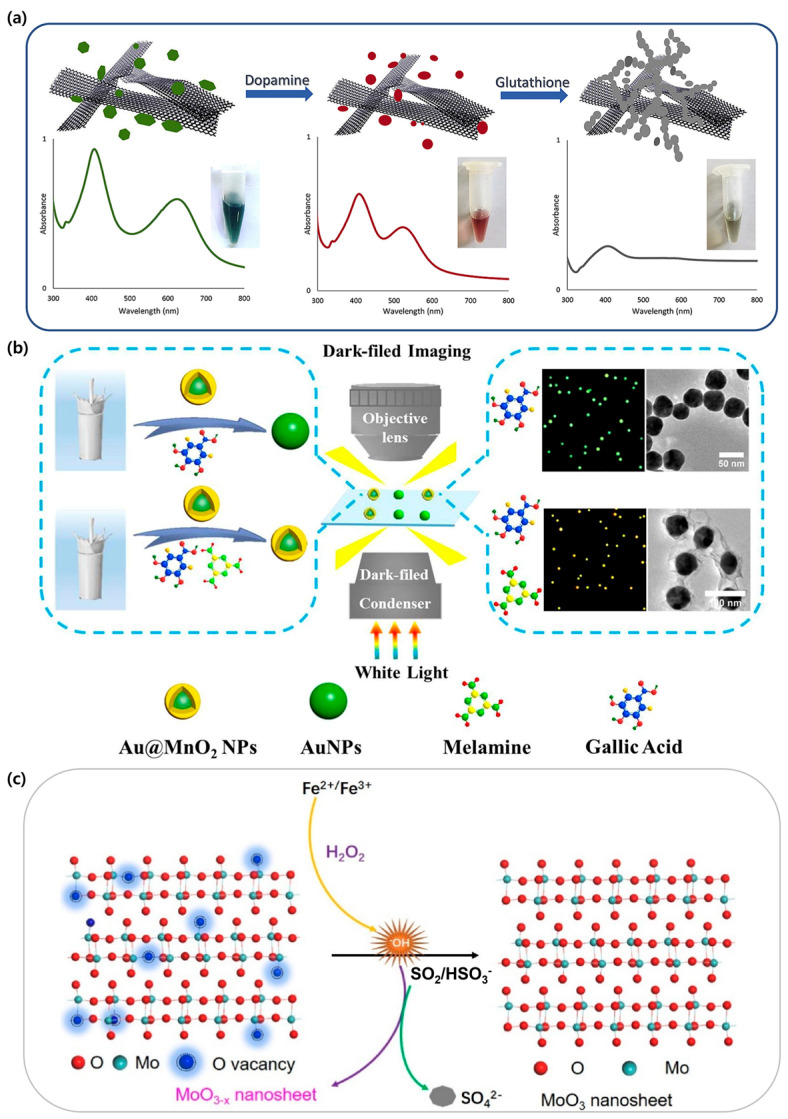
Noble metal decorated on a 2D-nanomaterial-based colorimetric sensing system. (**a**) Schematic illustrating the sequential colorimetric detection of DA and GSH using a GNR/AgNP hybrid sensor. Reproduced with permission from [132]. (**b**) Quantification of Mel-based Au@MnO_2_ NPs via the SPD method. The selectively etched MnO_2_ shell on the AuNPs surface results in distinctive color changes from a single Au@MnO_2_ NP. Reproduced with permission from [135]. (**c**) Colorimetric assays based on MoO_3−x_ nanosheets for the visual detection of atmospheric sulfate formation-involved substances. The blue MoO_3−x_ nanosheets turn colorless due to the oxidation of hydroxyl radicals (·OH) in the presence of Fe^2+^/Fe^3+^ and H_2_O_2_. Reproduced with permission from [140].

Due to their excellent properties, graphene-based materials find excellent applications in various biosensing and bioelectronics applications [130]. The study by Barghouti et al. shows the interaction of gold nanoparticles with a graphene film. The change in LSPR is shown to depend on the thickness of the graphene layer (ranging from 0.34 nm to 5 nm). It was confirmed that the resonance wavelength of SiOx/AuNPs/SiOx (574.71 nm) shifts to 657.90 nm when the graphene film is incorporated (SiOx/AuNPs/Graphene/SiOx) [131]. Rostami et al. proposed a novel plasmonic sensing platform that uses plasmon hybridization in a graphene nanoribbon/silver nanoparticle (GNR/AgNP) hybrid for the sequential colorimetric detection of dopamine (DA) and GSH. The DA etched the AgNPs, which caused a blue shift and a color change from green to red. In contrast, GSH induced the aggregation of AgNPs, which led to a color change from red to gray (Figure 4a). The LOD values for both DA and GSH were 0.46 μM and 1.2 μM, respectively, which highlights the effective role of GNRs in enhancing the sensitivity of the GNR/Ag NP hybrid [132].

Two-dimensional manganese dioxide (MnO_2_) nanosheets exhibit broad light absorption, a large surface area, excellent chemical stability, superior water solubility, low cost, and a straightforward synthesis [133]. Often, these 2D nanomaterials can be applied synergistically with metal nanoparticles. For example, Gao et al. proposed a novel approach to fabricate Au@MnO_2_ core–shell-assembled nanosheets utilizing LSPR in AuNPs. Their study demonstrated that the interaction between nanosheets and AuNPs can indeed modulate the plasmonic effect [134]. In addition, Yuan et al. developed a simple and label-free single-particle detection (SPD) method for the quantification of melamine (Mel) using MnO_2_-modified AuNPs (Au@MnO_2_ NPs) (Figure 4b). In the presence of Mel, gallic acid facilitated the degradation of the MnO_2_ shell, which resulted in a noticeable color change from yellow to green. The LOD achieved 16.7 nM (approximately 0.002 ppm), demonstrating satisfactory results of the proposed sensing system [135]. Zhang et al. studied a high-sensitivity plasmonic color measurement biosensor for exosome quantification using the etching of gold nanobipyramid@MnO_2_ nanosheet nanostructures (AuNBP@MnO_2_ NSs). Capturing exosomes with a capture probe activates ALP, which breaks down AAP to produce ascorbic acid (AA). The etching step of AA’s AuNBP@MnO_2_ NSs changed the refractive index of the AuNBPs and was accompanied by a blue shift of the longitudinal localized surface plasmon resonance peak. When etched, the color changed from brown to blue, and the LOD of the exosome was 1.35 × 10^2^ parts/μL [116].

Molybdenum trioxide (MoO_3_) nanosheets are both excellent and cost-effective 2D nanostructures with LSPR effects. The MoO_3−x_ nanosheets, which are partially oxygen-deficient due to reduction by AA, exhibit an LSPR absorption peak near 820 nm and appear blue. These doped MoO_3−x_ nanosheets also show LSPR effects and serve as promising alternatives to AuNPs and AgNPs [136,137,138,139].

Li et al. developed a colorimetric analysis method for Cu^2+^ based on the pH-dependent formation of plasmonic MoO_3−x_ nanosheets. When Cu^2+^ is introduced, it reacts with AA to generate hydrogen ions, which leads to the formation of abundant MoO_3−x_ nanosheets. This process increases the free carrier concentration and results in strong LSPR absorption. The LOD for Cu^2+^ was 0.8 nM [138]. The study by Yu et al. represented a series of colorimetric analysis methods based on plasmonic MoO_3−x_ nanosheets for the visual detection of substances related to atmospheric sulfate formation. Moreover, the blue-colored plasmonic MoO_3−x_ nanosheets are oxidized by hydroxyl radicals (OH) generated via the Fenton reaction between Fe^2+^/Fe^3+^ and H_2_O_2_, resulting in colorless MoO_3_ nanosheets and a significant change in absorbance (Figure 4c) [140]. This section focuses on the unique optical properties of two-dimensional materials and the LSPR phenomenon based on them applied to colorimetric biosensors, suggesting that this approach is a promising strategy in terms of improving the sensor sensitivity and expanding the application range. A detailed list of colorimetric biosensors with 2D material-based nanoparticles is presented (Table 3).

### 3.2. Nanozyme-Based Colorimetric Sensing

Nanozymes are nanosized materials equipped with enzyme-mimicking features that are often integrated into colorimetric sensing due to their intrinsic properties of stability as well as ease of operation [141,142,143]. Recently, nanozymes have been receiving a massive spotlight, especially on their use in the fields of biosensing, clinical diagnosis, drug delivery, small-molecule detection, and others, thanks to their capabilities of having large surface areas, ease of preparation and storage, as well as low-cost processing [104,144,145]. Nevertheless, when compared to natural enzymes such as HRP, nanozymes struggle with limitations, especially in their practical applications. In brief, nanozymes face problems such as low specificity and comparatively low activity, and they mimic only limited types of enzymes [91,104,146]. Hence, researchers have been exerting maximum efforts to fully comprehend the synthesis of nanozymes with the primary goal [144]. In this section, based on the mechanism of colorimetric signal generation using oxidation–reduction reactions, we will discuss recent studies of colorimetric biosensors using various nanozymes and 2D nanomaterials.

#### 3.2.1. Enzyme-Like Nanomaterial-Based Colorimetric Biosensors

**Figure 5:**
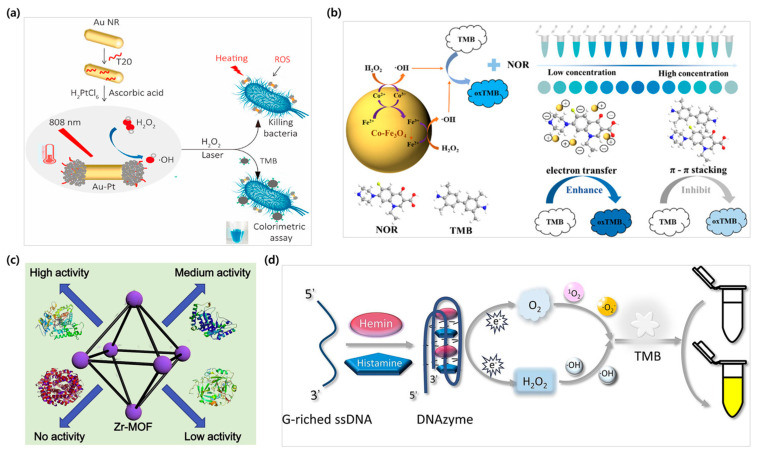
Enzyme-like nanomaterial-based colorimetric sensing. (**a**) Synthesis of anisotropic dumbbell-like Au-Pt nanoparticles with a DNA-encoded seed growth method for catalytic and anti-bacterial activities for bacterial detection. The DNA sequence polyT20 is regulated to form dumbbell-like Au-Pt bimetallic structures, while the synergy between Au and Pt results in high catalytic activity. Reproduced with permission from [159]. (**b**) Co-Fe_3_O_4_ nanozyme-based colorimetric sensing platform for NOR detection, showing color change intensity triggered by the increment and decrement of NOR concentration. The one-step hydrothermal synthesized Co-Fe_3_O_4_ MNP eliminates the optical interference as well as displays a remarkable peroxidase-like activity. Reproduced with permission from [163]. (**c**) Schematic illustrating Zr-based MOF with peroxidase activity. While Zr nodes interact specifically with phosphate groups in phosphorylated proteins, the dipyridyl-based ligands catalyze the TMB and H_2_O_2_ chromogenic reactions. Reproduced with permission from [141]. (**d**) Fabrication of DNAzyme via the insertion of hemin into a G-quadruplex DNA sequence. DNAzyme catalyzes H_2_O_2_ by dissolving oxygen, resulting in yellow products as the TMB molecules lose two electrons. Reproduced with permission from [168].

In the field of analytical chemistry, enzyme-based sensor techniques are categorized as one of the standard methods [141,147,148]. Nanozyme-based colorimetric sensing methods have been considered as assuring toolkit assays with a wide spectrum of biomarker detection [149,150], wherein the changeable physicochemical properties of nanozymes, especially their activity being controllable, render them attractive materials in the analytical fields [151,152,153,154,155]. Thus, to optimize the catalytic activity of nanozymes, their structure and shape must be continuously adjusted, and elemental doping is an effective approach to improve the catalytic performance by controlling the electronic structure and geometry [156]. Platinum (Pt) is a common nanozyme and is used in colorimetric biosensing research due to its high efficiency and stability in a wide range of pH and temperature conditions [157]. Fu et al. fabricated porous Au@Pt core–shell nanoparticles based on the excellent peroxidase-like activity of Pt nanoparticles for the detection of the SARS-CoV-2 virus [158]. In another study, Lu et al. constructed an Au-Pt bimetallic structure by growing Pt nanoparticles in a dumbbell shape on AuNR seeds using polyT20. Their system showed outstanding capabilities for both detecting and destroying *Escherichia coli* by utilizing the photothermal effect of AuNRs under light irradiation (Figure 5a). This study achieved a detection limit of 2 CFU/mL within 5 min [159].

Iron oxide (Fe_3_O_4_) nanoparticles are considered enzyme-like materials and have certain advantages over biosensor research, together with the fact that they are magnetic and capable of catalytic activation [160]. Since nanozymes can easily aggregate in water due to their low instability and dispersibility, the focus of a study was to modify an Fe_3_O_4_ nanozyme to maintain its properties [161]. Duan et al. produced Janus palladium (Pd)–Fe_3_O_4_ dumbbell nanoparticles and proposed a colorimetric assay to detect biothiols. As a result, the dispersion of the Janus Pd–Fe_3_O_4_ nanoparticles was very uniform and showed a higher catalytic oxidation effect when compared with the same amount of Pd and Fe_3_O_4_. This assay was also successfully applied to detect biothiols in real urine samples and showed an LOD of 3.1 nM in an aqueous solution [162]. Additionally, Nie et al. proposed a stable and sensitive smartphone color-measurement system based on cobalt-doped Fe_3_O_4_ magnetic nanoparticles (Co-Fe_3_O_4_ MNPs) for the visual detection of norfloxacin (NOR). Compared to Fe_3_O_4_, Co-Fe_3_O_4_ MNPs obtained a more markedly peroxidase-like activity. A low concentration of NOR accelerated the color reaction of TMBs (to darken the blue color of the solution), but at high concentrations, the nanozyme activity was suppressed, and the color gradually faded (Figure 5b). Based on this, a colorimetric sensor integrated with a smartphone RGB mode was developed with a visual detection limit of 0.08 µM [163].

Another example of nanozyme-like materials is metal–organic framework nanoparticles (MOFs). MOFs are highly porous materials that feature a high surface area and porosity and are commonly used for various applications, such as gas adsorption and separation, as well as drug carriers for controlled release. Various metals and ligands can be combined to implement the desired functions with these composite hybrid catalyst MOFs, and they are often used in sensing field applications [164]. Liu et al. implemented a Zn-MOF doped with Eu^3+^ ions and proposed a DEM fluorescence visual sensing platform using it. This study demonstrates the expansion from fluorescence sensors to quantitative and static visualization sensing platforms by utilizing the design flexibility of MOFs [165]. In a study conducted by Shi et al., a copper-based MOF (Cu-MOF) colorimetric assay was developed for the selective identification of *Alicyclobacillus acidoterrestris* and the detection of guaiacol. The enzyme-like nanomaterials were fabricated by allowing 4,4′-bipyridine and Cu ions to self-assemble at room temperature.

Furthermore, MOFs play a role in mimicking peroxidase for the detection of *A. acidoterrestris* and achieve a highly selective detection of guaiacol as compared to other polyphenols with a high sensitivity of the LOD of 0.003 mM. The peroxidase-like activities of MOFs have been extensively applied for the reduction of compounds such as glucose, AA, and H_2_O_2_, yet this study is the first attempt to utilize MOFs for the detection of guaiacol and *A. acidoterrestris* [166]. Wang et al. conducted research on zirconium-based MOFs (Zr-MOFs), which were synthesized as peroxidase mimics to catalyze the chromogenic reaction of H_2_O_2_ and 3,3′,5,5′-tetramethylbenzidine for the detection and quantification of phosphorylated proteins, with α-casein (α-CS) as the target protein (Figure 5c). Specifically, the Zr nodes in the MOF provide specific sites for recognizing phosphate groups in proteins, which enables simple yet efficient quantification of α-CS with an LOD of 0.16 µg/mL and a range from 0.17 to 5.0 µg/mL [141].

From the perspective of nanomaterial-based biosensors, a DNAzyme consists of a guanine (G)-rich sequence with hemin as a monomer, enclosed in a G-quadruplex structure, which forms the foundation of the DNAzyme with robust peroxidase activity. This property has made DNAzymes a hotspot in the sensing field due to their noteworthy qualities [167]. DNAzymes are reported to be highly stable under heat treatments, resistant to hydrolysis, easy to label, and cost-effective, further promoting their incorporation into the assembly of colorimetric biosensors [168,169]. However, most DNAzyme-based sensing systems in complex biological samples rely on inhibiting enzymatic catalytic activities, which can lead to false-positive results [168]. Thus, Wang et al. carried out a study where they discovered that the incorporation of histamine can increase the catalytic activity of DNAzymes. In summary, the presence of histamine encourages G-quadruplex sequence generation, allowing for effortless bonding with hemin and fabricating many DNAzyme molecules (Figure 5d). The performance of the proposed biosensor in terms of sensitivity demonstrates a low LOD of 38 µg/L for histamine with anti-interference ability, high selectivity, and a good recovery rate in actual meats, which creates opportunities for the pre-evaluation of freshness and histamine content of real meat products [168].

Furthermore, heterostructured nanomaterial-based nanozymes, such as copper sulfide/zinc sulfide (CuS/ZnS), are constructed from a single component and function as multifaceted nanobiological sensors. Their outstanding capability and synergistic performance arise from the coordination of nanomaterials [149,170]. Tian et al. fabricated a CuS/ZnS nanozyme plasmon-stimulated colorimetry biosensor array, wherein ZnS was packaged in hollow CuS nanocubes. This enabled the regulation of the LSPR effect, along with a remarkable peroxidase-like catalytic activity for the detection of three metabolites.

The proposed biosensor system achieved an LOD as low as 1 µM for each of the target metabolites: cysteine (Cys), AA, and GSH. Each metabolite can be selectively detected both qualitatively and quantitatively using a principal component analysis within the range of 5 to 1000 µM. This biosensor system is both capable and robust, and it is hoped that it will contribute to the development of sensors in the field of clinical detection [149]. In this section, various studies on nanozyme-based colorimetric biosensors were discussed, highlighting their enzyme-mimicking activity, which enables enhanced stability, cost-effectiveness, and signal amplification, demonstrating their potential as next-generation diagnostic platforms. A detailed list of colorimetric-based biosensors with nanozymes is presented (Table 4).

#### 3.2.2. Enzyme-Mimicking 2D Nanomaterial-Based Biosensors

In this section, catalytically active nanomaterials with a 2D structure are reviewed in-depth. Currently, 2D-structured nanozymes are highly valued due to their distinctive physical and chemical properties, such as a wide surface area, excellent biocompatibility, electrical conductivity, and catalytic activity, along with ease of fabrication and modification [171,172]. Due to these unique characteristics, various types of 2D nanomaterials are being explored, which advances research across various fields.

Ti_3_C_2_T_x_ MXene has gained significant attention due to its excellent intrinsic properties, such as metallic conductivity, superior hydrophilicity, and good stability [173]. The terminal groups of MXene contribute to the regulation of the oxidation state of Ti atoms, which facilitates the electron transfer. Wang et al. utilized oxygen-terminated few-layer titanium-based MXene nanosheets (OFL-Ti-MNs) for the detection of kanamycin (KAN). The Ti_3_AlC_2_ MAX phase material underwent chemical etching using a LiF/HCl-based method to remove the aluminum (Al) layer, which resulted in the synthesis of an oxygen-terminated multilayer MXene with various functional groups, such as –O, –OH, and –F, on its surface. In the presence of KAN, the TMB oxidation reaction is inhibited, preventing the expected color change. This analysis required less than 15 min and provided an LOD of 15.3 nM for KAN [174].

**Figure 6:**
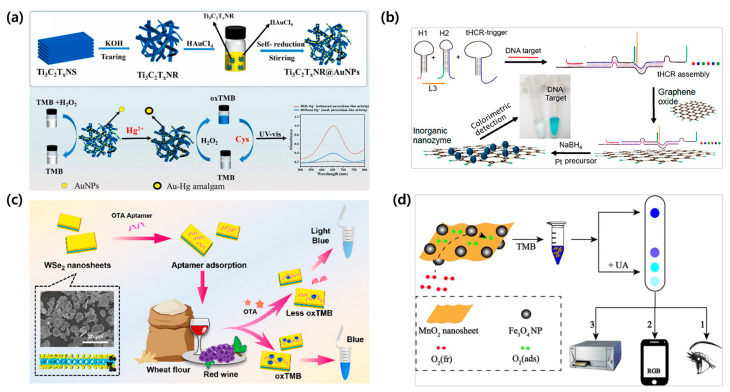
Enzyme-mimicking 2D nanomaterial-based colorimetric sensing. (**a**) Preparation of Ti_3_C_2_T_x_NR@AuNPs nanohybrids and the mechanism for nanozyme colorimetric detection of Hg^2+^ and Cys based on Ti_3_C_2_T_x_NR@AuNPs. Reproduced with permission from [175]. (**b**) Schematic illustrating a DNA-controlled approach and growth of Pt nanoparticles on GO–PtNPs for the colorimetric detection of mutant KRAS. GO supports the inorganic nanozyme formation by PtNPs in situ growth on its surface. Reproduced with permission from [177]. (**c**) Illustration of label-free WSe_2_ nanosheet synthesis for OTA detection via a colorimetric aptasensor. Reproduced with permission from [178]. (**d**) Biomimetic oxidase-based colorimetric determination, integrating Fe_3_O_4_@MnO_2_ for UA quantification. Reproduced with permission from [151].

Zhou et al. constructed a platform for the high-sensitivity colorimetric detection of mercury ions by fabricating Ti_3_C_2_T_x_ MXene nanoribbons decorated with AuNPs (Figure 6a). The peroxidase-mimicking activity of the Ti_3_C_2_T_x_ MXene nanoribbons against TMB was enhanced in the presence of mercury ions, which resulted in the oxidation of TMB and the color of the solution changing from colorless to dark blue. The achieved LOD was 0.054 nM, which was significantly lower than the critical level (about 10.0 nM) of mercury ions in drinking water, as defined by the U.S. Environmental Protection Agency [175].

Moreover, progress has advanced sufficiently for the use of graphene oxide (GO) in engineering, nanocomposites, biosensors, and drug delivery. In particular, GO exhibits peroxidase-like activity and is used in colorimetric biosensors [176]. For instance, Chen et al. revealed that, due to its amphiphilicity, wide surface area, and distinct affinities for ssDNA and dsDNA, GO facilitates the in situ growing of inorganic nanozyme Pt nanoparticles (PtNPs) on its surface. The integration of DNA-controlled growth of PtNPs and target DNA-triggered triplex-hybridization chain reaction (tHCR) on GO regulates the nanozyme activity, which enables the fabrication of nanozyme-catalyzed colorimetric biosensors (Figure 6b). Accordingly, superior sensitivity for mutant KRAS DNA and let-7a microRNA as model targets was achieved with an LOD down to 14.6 pM and 21.7 pM, respectively. The proposed system displayed transferability that can be used for other nucleic acid targets and integration with different inorganic nanomaterials [177].

In addition, graphene-like nanosheets of TMDs, such as tungsten disulfide (WS_2_) and tungsten diselenide (WSe_2_), have also been reported for their intrinsic peroxidase-mimicking activity, and they are commonly integrated into colorimetric detection methods [178,179]. A study by Zhou et al. reported that the conjugation of an aptamer on layered WS_2_ nanosheets has been profoundly enhancing the peroxidase-mimicking activity of nanosheets. Specifically, a straightforward and stable colorimetric aptasensor for KAN recognition was fabricated with an LOD as low as 0.06 µM within a linear range of 0.1 to 0.5 µM, exhibiting great selectivity against other tested antibiotics [179]. In another recent study, 2D few-layer WSe_2_ nanosheets were fabricated with an aptamer to quantify ochratoxin A (OTA) (Figure 6c). Correspondingly, the adsorbed OTA aptamer on the WSe_2_ nanosheet induced an enhanced enzymatic catalytic property of the nanosheet, with a good linear relationship of R^2^ = 0.99 with 0.16 ng/mL of the LOD in the range of 0.5 to 50 ng/mL of OTA [178].

MnO_2_-based nanozymes offer the advantages of having good biocompatibility, low processing cost, and rapid and simple handling in catalytic reactions [105]. Yang et al. fabricated Fe_3_O_4_@MnO_2_ nanosheet-based colorimetric biosensors for the detection of uric acid (UA) through three different visualization methods: naked eye, UV–vis spectrophotometry, and smartphone colorimetry (Figure 6d). In short, thanks to Fe_3_O_4_ doped in between the MnO_2_ nanosheets, the specific surface area increased, which further cleared the space and path for electron conduction and oxidation and, thus, enhanced the catalytic activity of the MnO_2_ nanosheet. As a result, the LOD values for UA were 0.27 µM and 21 µM through UV–vis spectrometry and smartphone-based colorimetry, respectively, with linear ranges of 1 to 70 µM and 200 to 650 µM [151]. In light of these findings, it is evident that nanosheets coordinate the activities of nanozymes, thus allowing sensors to be fabricated using aptamer adsorption. This section reviewed studies on 2D nanomaterial-based colorimetric biosensors utilizing redox reactions, demonstrating their potential for diagnostic applications. A detailed list of colorimetric-based biosensors with 2D material-based nanozymes is presented (Table 5).

## 4. The Fusion of Colorimetric and Other Analytical Techniques

Colorimetric biosensors are based on sensing techniques that produce color changes through specific biochemical reactions with target analytes [180]. Unfortunately, there is a limit to observing color changes with the naked eye; therefore, more precise analytical methods are needed to accurately confirm the output signals. Several advanced techniques have been developed to address this limitation. For example, to increase the detection sensitivity by combining colorimetric signals with other signals, a device-based analysis that can quantitatively measure color changes and data analysis techniques using AI and machine learning was assembled in one platform [181,182,183]. As the analysis of colorimetric sensors using smartphones continues to be actively studied, the development of a highly portable diagnostic platform is also gaining attention in the sensing field. Specifically, the adaptation of the latest technologies, such as machine learning in colorimetric biosensors, makes it possible to analyze even a minor color change accurately, which improves the diagnostic accuracy of biosensors [32]. In this section, we would like to comprehensively discuss the application of colorimetric biosensors, especially the principles and detection techniques of the latest biosensing platform technologies.

### 4.1. Dual-Mode Sensing Technologies

**Figure 7:**
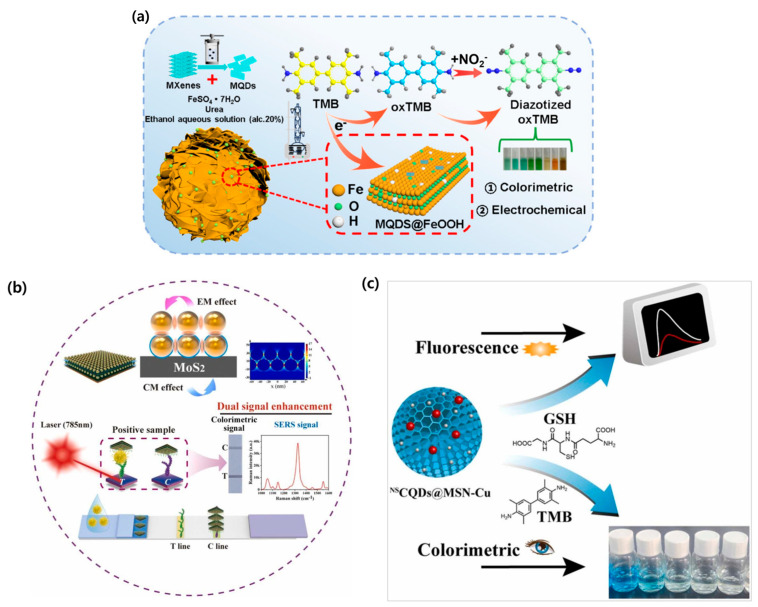
Biosensors incorporating dual-mode detection techniques. (**a**) Dual-mode assay combining colorimetric and electrochemical methods for the determination of nitrite using MQDs@FeOOH nanozymes. Reproduced with permission from [190]. (**b**) Dual-signal enhancement LFA integrating MoS_2_-based colorimetric and SERS for MPXV detection. Reproduced with permission from [193]. (**c**) Schematic illustrating a dual-mode sensor (NSCQDs@MSN-Cu) with fluorescence and colorimetric detection of GSH. Reproduced with permission from [198].

The main advantage of colorimetric biosensors is that they enable rapid detection by visually observing color changes, thus making them highly suitable for POCT and routine monitoring [184]. However, a simple visual observation alone is insufficient for detecting low concentrations of target analytes. To overcome this limitation, approaches incorporating detection techniques such as electrochemical, Raman, and fluorescence methods have been developed [185,186,187]. For example, electrochemical methods utilize analysis techniques like potentiometry, amperometry, and impedance to detect a charge transfer at an electrode when the target interacts with it. This approach offers high sensitivity and the benefit of real-time monitoring [188]. Leveraging these benefits, Zhang et al. proposed an electrochemical–colorimetric dual-mode biosensor for the effective detection of cardiac troponin I (cTnI). The group utilized aptamer-functionalized Fe^3+^-polydopamine (Apt@Fe^3+^-PDA) nanomaterials, in which, upon binding to cTnI under acidic conditions, Fe^3+^ was released, which led to its conversion into Prussian Blue and the subsequent generation of both electrochemical and colorimetric signals. The LOD values achieved through colorimetric and electrochemical methods were 7.4 pg/mL and 3.2 pg/mL, respectively, which indicates ultrasensitive detection [189]. Similarly, Zhan et al. developed a dual-mode biosensor using FeOOH nanostructures modified with MXene quantum dots to effectively detect nitrite (NO_2_^−^) (Figure 7a). The active site of the Fe-Ti heterodimer decomposes H_2_O_2_, generating reactive oxygen species (•OH) that react with nitrite to produce both a color output and an electrochemical signal. The LOD values for nitrite detection were 1.58 μM (colorimetric) and 2.99 μM (electrochemical), demonstrating the potential of this sensor platform for next-generation environmental monitoring applications [190].

Next, Raman spectroscopy is a technique that identifies specific substances based on their unique Raman signals. It is widely applied in various biosensor applications [191]. However, Raman signals are inherently weak, which requires the use of surface-enhanced Raman scattering (SERS) to amplify them [192]. Since SERS signals are highly sensitive to the distance between molecules and nanomaterials, integrating SERS with colorimetric biosensors can compensate for the low sensitivity of colorimetric detection. Yu et al. developed an SERS-colorimetric-based immunochromatography assay for the effective detection of the monkeypox virus (MPXV). This assay rapidly identified the target virus using colorimetric signals while enabling the detection of low-concentration targets through SERS signals (Figure 7b). The LOD values for colorimetric and SERS detection were 0.2 ng/mL and 0.002 ng/mL, respectively, exhibiting a performance that was 5 to 500 times superior to conventional immunochromatographic assays (ICA) [193]. Additionally, Sun et al. developed a SERS/colorimetric-based biosensor for detecting GSH, a biomarker for the early diagnosis of cancer and Parkinson’s disease in human blood. By using AuNPs@Cu-porphyrin MOFs, the group observed both SERS and colorimetric outputs, achieving LOD values of 5 nM and 1 μM and demonstrating ultrasensitive detection [194].

Fluorescence occurs when molecules absorb light and subsequently emit it at a different wavelength. Fluorescence-based sensing techniques enable highly sensitive and non-destructive analyses [195]. Recently, increasing research has been conducted on dual sensors that combine fluorescence with colorimetric detection. Fluorescence–colorimetric dual-mode sensors offer the benefits of rapid detection times and more sensitive detection compared to conventional sensors [196]. For instance, Wang et al. developed a dual-signal ICA combining fluorescence and colorimetric signals for monkeypox virus detection, achieving an LOD of 0.00024 pg/mL and 0.1 ng/mL, respectively, which is 10 to 400 times more sensitive than conventional ICA methods [197]. Additionally, Li et al. developed a fluorescence–colorimetric dual sensor that showed highly sensitive detection of GSH, a biomarker for diabetes and neurodegenerative diseases, with an LOD of 1.6 μM and 7 μM, respectively (Figure 7c) [198]. This section reviewed dual-mode biosensor studies combining colorimetry with SERS, electrochemical, and fluorescence techniques, highlighting their contribution to enhanced sensitivity and diagnostic reliability through multimodal analyses. A detailed list of colorimetric-based dual-mode biosensors is presented (Table 6).

### 4.2. AI and Machine Learning-Assisted Colorimetric Biosensors

Colorimetric results are difficult to interpret accurately with the naked eye because subtle color changes can be affected by environmental factors such as lighting conditions and visual variation. As a result, various methods that leverage different instruments have been studied to reliably observe color changes, recently generating significant research interest in smartphone-based colorimetric detection methods [199,200,201,202]. As an example, Choi et al. developed a fast and reliable smartphone-assisted colorimetric biosensor. This smartphone-assisted colorimetric biosensor showed high selectivity with respect to various interfering substances in human urine. The LOD for a urine solution was 0.0036 mM, while 0.58 mM was achieved for urine detected on paper, which indicates high sensitivity [106].

Moreover, Zhao et al. developed a smartphone-enabled colorimetric biosensor that quickly distinguishes between a variety of pesticides within a real sample. Glyphosate, tyram, imidacloprid, and five other pesticides yielded LOD values of 1.5 × 10^−7^ M—a concentration that is much lower than the one required by the conventional U.S. Environmental Protection Agency (1.18 × 10^−6^ to 3.91 × 10^−6^ M). The group demonstrated a significant improvement in accuracy and detection speed by analyzing the RGB color changes in smartphones resulting from AuNPs aggregation [203].

**Figure 8:**
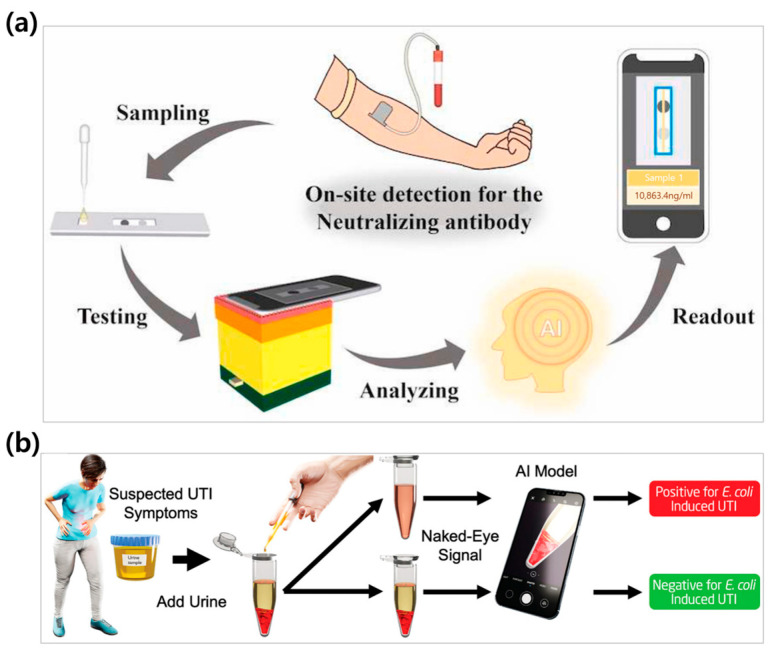
AI and machine learning integration in colorimetric biosensors. (**a**) AI-assisted colorimetric PDA-LFIA platform for the sensitive and accurate quantification of neutralizing antibodies from vaccinations. Reproduced with permission from [209]. (**b**) Schematic diagram showing the potential use of a CNN detection platform for UTI detection at home. Reproduced with permission from [211].

With recent advancements in science and technology, AI and machine learning algorithms are becoming more sophisticated, introducing revolutionary changes to the field of biosensors [32,204]. Although colorimetric biosensors have the benefit of rapid target detection, they suffer from low sensitivity. Therefore, by integrating AI and machine learning into colorimetric biosensors, it becomes possible to detect subtle color changes that are difficult to distinguish for the human eye. This technology can further improve the sensitivity by differentiating not only color changes but also saturation and intensity, thereby allowing the distinction of even lower LOD values [205,206,207]. Ouyang et al. researched the effective detection of volatile organic compounds (VOCs) using AI and colorimetric biosensors. The group developed an AI technology that mimics human olfactory functions and measures color changes, which enabled the detection of VOCs at sub-ppm levels through direct visualization and monitoring [208]. Similarly, Tong et al. combined colorimetric lateral flow assays (LFAs) with AI to detect COVID-19 disease-neutralizing antibodies. The AI analysis algorithm evaluated the color changes in the control and test lines formed through antigen–antibody reactions (Figure 8a). Consequently, this method provided faster and more accurate results compared to conventional enzyme-linked immunosorbent assay (ELISA) LFA kits and allowed for the detection of extremely low concentrations of target antibodies (with an LOD of 160 ng/mL) [209].

Machine learning has been actively utilized to improve the performance of colorimetric-based biosensors [210]. Mann et al. developed a colorimetric DNAzyme cross-linked hydrogel sensor to detect *Escherichia coli* and paired it with an AI model to eliminate ambiguity in sensor readings (Figure 8b). In real-life lake water samples, *E. coli* was detected at a concentration of 10^1^ CFU/mL, which suggests the potential for broad applications for the detection of other bacteria. The researchers trained a convolutional neural network (CNN) AI model with optical images, further automating and enhancing the usability of this platform. With this machine learning platform, they detected urinary tract infections (UTI) in real patient urine samples (that were automatically readable) with more than 96% accuracy [211]. Subsequently, Hassani-Marand et al. proposed a machine learning platform for detecting catecholamine neurotransmitters (CNs), such as dopamine (DA), epinephrine (EP), norepinephrine (NEP), and levodopa (LD), in human urine. These are associated with Parkinson’s and Alzheimer’s disease. The different aggregation behaviors of AuNPs generated unique fingerprint response patterns, which were input to the platform to classify samples automatically. The linear discriminant analysis (LDA) algorithm was used to distinguish between the different neurotransmitters, while partial least squares regression (PLSR) was employed for the quantitative concentration analysis [212]. Therefore, by integrating these chemical color change principles with nanotechnology, AI, and smartphone-based analytical systems, colorimetric biosensors can be further developed into more precise and user-friendly diagnostic systems, opening the path for a new paradigm in diagnostics [213]. A detailed list of instrument-assisted colorimetric biosensors is presented (Table 7).

## 5. Conclusions and Perspectives

This review discussed the fundamental principles, recent advances, and practical applications of colorimetric biosensors based on nanomaterials. These biosensors offer notable advantages, such as simplicity in visual detection, low cost, rapid analysis, portability, and suitability for POCT, making them attractive tools in diverse fields, including disease diagnostics, environmental monitoring, and food safety testing.

The integration of various nanomaterials such as noble metal nanoparticles and 2D materials like graphene, WS_2_, MnO_2_, and nanozymes has significantly improved the sensitivity, selectivity, and reliability of colorimetric biosensors. Particularly, gold nanoparticles (AuNPs) have been extensively used due to their tunable optical properties, while nanozymes and 2D nanomaterials contribute to signal amplification through enhanced catalytic activities. Furthermore, with the aid of smart devices, machine learning, and AI-driven data processing, the limitations of traditional colorimetric detection, such as poor quantifiability and subjectivity, are being effectively addressed, promoting the development of portable and intelligent sensor systems.

Additionally, technological advances have led to the integration of smartphones and artificial intelligence in colorimetric analyses. RGB-based signal interpretation using smartphone cameras, combined with machine learning algorithms, allows for automated, quantitative analyses with reduced user variability. These smart systems can process multiple signal patterns, enabling real-time monitoring and accurate decision-making, thus enhancing the commercial viability of portable colorimetric biosensors.

However, despite these technological advances, the clinical translation of nanomaterial-based colorimetric biosensors remains challenging. Most studies are still confined to laboratory-scale optimization and do not fully account for the complexity of real biological environments. Issues such as reduced reproducibility in clinical samples, limited long-term stability, and potential biological interference from the nanomaterials themselves persist. Standardized clinical validation protocols tailored to nanomaterial-based biosensors are still lacking, with most studies limited to in vitro or animal model testing [33,34,35,36,37]. Moreover, large-scale synthesis is hindered by their inherent instability and sensitivity to environmental conditions, resulting in increased production costs and difficulties in standardization [39,40]. Also, a lack of active collaboration between material scientists and clinical practitioners further contributes to a gap between sensor design and real-world medical needs [38].

To overcome these barriers, it is essential to establish a full-chain collaborative model integrating basic research, sensor development, and clinical evaluation. The active involvement of clinicians from the early stages of sensor design to the final stages of validation will be crucial in translating these promising technologies into practical clinical tools. Future efforts should prioritize resolving real-world implementation issues, thereby promoting the successful adoption of nanomaterial-based colorimetric biosensors in clinical practice.

## Figures and Tables

**Table 1:** Principles of colorimetric-based biosensors with their main influencing factors.

Principles	Key Influencing Factors
LSPR-based Peak shift upon target binding Color change via redox reaction	1. Noble metal (Au and Ag) 2. Size and shape 3. Aggregation 4. Local environment (binding events, pH)
Redox-based Color change via redox reaction	1. Enzyme activity 2. Colorimetric substrate 3. Redox mediator presence (nanozymes) 4. Analyte concentration

**Table 2:** List of colorimetric-based biosensors with noble metal-based nanoparticles.

Principle	Strategy	Target	Probe	LOD	Mechanism	Reference
LSPR	Noble metal-based nanoparticles	*Salmonella typhimurium*	Au	3.2 × 10^3^ CFU/mL	LSPR effect by Au aggregation	[118]
		biothiol	AgNPR	0.041 µM	LSPR effect by AgNPR aggregation	[120]
		NH_3_	Au@Ag@AgCl core–shell nanoparticles	6.4 µM	LSPR effect by noble metal-core exposure	[125]
		*Escherichia coli* O157:H7	BUHNPs	2.48 × 10^3^ CFU/mL	LSPR effect by changes in size and shape	[126]

**Table 3:** List of colorimetric-based biosensors with 2D material-based nanoparticles.

Principle	Strategy	Target	Probe	LOD	Mechanism	Reference
LSPR	2D materials-based nanoparticles	DA GSH	GNR/AgNP	0.46 μM 1.2 μM	LSPR effect by changes in AgNPs	[132]
		Mel	Au@MnO_2_ NPs	16.7 nM	LSPR effect by local environment	[135]
		Exosomes	AuNBP@MnO_2_ NSs	1.35 × 10^2^ particles/μL	LSPR effect by local environment	[116]
		Cu^2+^	MoO_3−x_ nanosheet	0.8 nM	LSPR effect by local environment	[138]
		H_2_O_2_ Fe^2+^ Fe^3+^ HSO_3_^−^ SO_2_	MoO_3−x_ nanosheet	60 nM 50 nM 400 nM 1 μM 50 ppb	LSPR effect by local environment	[140]

**Table 4:** List of redox-based colorimetric biosensors with nanozymes.

Principle	Strategy	Target	Probe	LOD	Mechanism	Reference
Redox	Nanozymes	SARS-CoV-2	Au@Pt core–shell NPs	11 ng/mL	Enzyme activity of Au@Pt NPs	[158]
		*Escherichia coli* O157:H7	Au@Pt dumbbell NPs	2 CFU/mL	Enzyme activity of Au and Pt	[159]
		biothiol	Janus Pd–Fe_3_O_4_ dumbbell NPs	3.1 nM	Enzyme activity of Janus Pd-Fe_3_O_4_	[162]
		NOR	Co-Fe_3_O_4_ MNP	0.08 µM	Enzyme activity of Co-Fe_3_O_4_ MNP	[163]
		*Alicyclobacillus acidoterrestris*	Cu-MOF	0.003 mM	Enzyme activity of Cu-MOF	[166]
		α-CS	Zr-MOF	0.16 µg/mL	Enzyme activity of Zr-MOF	[141]
		histamine	DNAzyme	38 µg/L	Enzyme activity of DNAzyme	[168]
		Cys AA GSH	CuS/ZnS	1 µM (each)	Enzyme activity of CuS/ZnS	[149]

**Table 5:** List of redox-based colorimetric biosensors with 2D material-based nanozymes.

Principle	Strategy	Target	Probe	LOD	Mechanism	Reference
Redox	2D materials-based nanozymes	KAN	OFL-Ti-MN	15.3 nM	Changes in substrate concentrations	[174]
		Hg^2+^	Ti3C2TxNR@AuNPs nanohybrids	0.054 nM	Changes in substrate concentrations	[175]
		KRAS DNA let-7a	GO-PtNP	14.6 pM 21.7 pM	Enzyme activity of GO-PtNP	[177]
		KAN	aptamer- enhanced WS_2_ nanosheets	0.06 µM	Presence of redox mediator	[179]
		OTA	aptamer- enhanced WSe_2_ nanosheets	0.16 ng/mL	Presence of redox mediator	[178]
		UA	Fe_3_O_4_@MnO_2_ nanosheets	0.27 µM	Enzyme activity of Fe_3_O_4_@MnO_2_	[151]

**Table 6:** List of colorimetric-based dual-mode biosensors.

Dual Mode	Target	Probe	LOD	Reference
Colorimetric–electrochemical	cTnI	Apt@Fe^3+^-PDA	7.4 pg/mL (colorimetric) 3.2 pg/mL (electrochemical)	[189]
	NO_2_^−^	MQDs@FeOOH	1.58 μM (colorimetric) 2.99 μM (electrochemical)	[190]
Colorimetric–SERS	MPXV	MoS_2_@Au–Au	0.2 ng/mL (colorimetric) 0.002 ng/mL (SERS)	[193]
	GSH	AuNPs@Cu-porphyrin MOF	1 μM (colorimetric) 5 nM (SERS)	[194]
Colorimetric–fluorescence	MPXV	MoS_2_-TQD	0.1 ng/mL (colorimetric) 0.00024 pg/mL (fluorescence)	[197]
	GSH	CQDs@MSN-Cu	7 μM (colorimetric) 1.6 μM (fluorescence)	[198]

**Table 7:** List of instrument-assisted colorimetric biosensors.

Sensing Platform	Target	Probe	LOD	Reference
Smartphone-assisted	Urea	AgNPs	0.58 mM	[106]
	Glyphosate Thiram Imidacloprid Tribenuron methyl Nicosulfuron Thifensulfuron methyl Dichlorprop Fenoprop	AuNPs	1.5 × 10^−7^ M (each)	[203]
AI-assisted	VOCs	Dye@ZIF-8@COF	<1 ppm	[208]
	COVID-19 neutralizing antibodies	PDA@polymer^3^@SiO_2_@PEG-RBD	160 ng/mL	[209]
	*Escherichia coli*	DNAzyme	10^1^ CFU/mL	[211]
	DA EP NEP LD	AuNPs	0.3 μM 0.5 μM 0.2 μM 1.9 μM	[212]

## Data Availability

Not applicable.
